# Relative Validity of a Method Based on a Smartphone App (Electronic 12-Hour Dietary Recall) to Estimate Habitual Dietary Intake in Adults

**DOI:** 10.2196/11531

**Published:** 2019-04-11

**Authors:** Luis María Béjar, María Dolores García-Perea, Óscar Adrián Reyes, Esther Vázquez-Limón

**Affiliations:** 1 Department of Preventive Medicine and Public Health School of Medicine University of Seville Seville Spain; 2 Virgen Macarena University Hospital Seville Spain; 3 Mutua Balear Seville Spain

**Keywords:** epidemiologic methods, diet records, mobile apps, nutrition assessment

## Abstract

**Background:**

Accurate dietary assessment is key to understanding nutrition-related outcomes and for estimating the dietary change in nutrition-based interventions. When researching the habitual consumption of selected food groups, it is essential to be aware of factors that could possibly affect reporting accuracy.

**Objective:**

This study aimed to evaluate the *relative validity* of the current-day dietary recall, a method based on a smartphone app called electronic 12-hour dietary recall (e-12HR), to categorize individuals according to habitual intake, in the whole sample of adults and in different strata thereof.

**Methods:**

University students and employees over 18 years recorded the consumption of 10 selected groups of food using e-12HR during 28 consecutive days. During this period, they also completed 4 dietary records. Once the period was finished, the subjects then completed a food frequency questionnaire (FFQ) and a usability-rating questionnaire for e-12HR. The food group intakes estimated by the e-12HR app, the dietary records, and the FFQ were categorized into sextiles: *less than once a week, once or twice a week, 3-4 times a week, 5-6 times a week, once or twice a day,* and *3 or more times a day*. The 10 selected groups with e-12HR were compared with 4 dietary records and an FFQ reference method, in the whole sample and in different strata thereof: age (years): <25 and ≥25; gender: females and males; occupation: students and employees; smoking: no and yes; physical activity (minutes/week): ≥150 and <150; and body mass index (kg/m^2^): <25 and ≥25. The association between the different methods was assessed using Spearman correlation coefficient (SCC). Cross-classification and kappa statistic were used as a measure of agreement between the different methods.

**Results:**

In total, 203 participants completed the study (56.7% [115/203] women, and 43.3% [88/203] men). For all food groups and all participants, the mean SCC for e-12HR versus FFQ was 0.67 (≥0.62 for all strata). On average, 50.7% of participants were classified into the same category (≥47.0% for all strata) and 90.2% within the nearest category (≥88.6% for all strata). Mean weighted kappa was 0.49 (≥0.44 for all strata). For e-12HR versus RDs, mean SCC was 0.65 (≥0.57 for all strata). On average, 50.0% of participants were classified into the same category (≥47.0% for all strata) and 88.2% within the nearest category (≥86.1% for all strata). Mean weighted kappa was 0.50 (≥0.44 for all strata).

**Conclusions:**

The results indicate that e-12HR generated categories of dietary intake highly comparable with the 2 reference methods in the whole sample and in different strata thereof. The inclusion of photographs to facilitate estimation of the servings consumed generated correlation/agreement data between e-12HR and the FFQ that were similar to a previous study using an older version of the app, which did not include photographs.

## Introduction

### Background

Habitual intake (or average long-term consumption) is an essential part of epidemiological investigations and intervention studies [1-3]. Many of these studies do not require the characterization of all foods and beverages consumed (hereafter referred to as *food*) [4], as it can represent an unnecessary workload for study participants and an avoidable waste of the scarce resources available for research [5]. The characterization of foods may mean assessing whether survey items can be reduced to binaries (was a food eaten or not?) or requiring an accurate weight [6]. Categorizing individuals according to categories of habitual consumption of specific food groups might be used for evaluating the relationship between relative ranking and disease [2,7-13] and for evaluating the effectiveness of personalized methods that are implemented to promote changes in dietary patterns [2,4,8-11] with regard to the selected food groups.

Dietary records (DRs) and 24-hour recalls (short-term methods), and food frequency questionnaires (FFQs; a long-term method) are the 3 main assessment methods that are traditionally used to assess dietary intake [14-16]. The strengths and weaknesses of these instruments are well documented [2,7,14,17-20].

In large-scale epidemiological and intervention studies, where detailed dietary assessment is not feasible [9], FFQs have been the most accessible and commonly utilized dietary assessment tool [1,7,10,11,15,16,21]. FFQs are retrospective methods that require respondents to report the frequency of consumption of a predefined list of food groups over an extended period of time (weeks or months) [14,22]. FFQs are practical and easy to administer; they do not affect food intake patterns and can assess habitual dietary patterns with a single administration [10]. One inherent limitation to most FFQs is that they are paper-based. As a result, on the one hand, errors such as skipped questions or multiple marks are common, whereas on the other hand, they do not allow precise estimation of food portion size [8], and finally, there is the necessary posterior manual introduction of data for statistical analysis, which increases research costs and time consumption considerably [19,23]. FFQs in digital format (mobile phone apps or Web-based) offer straightforward solutions to these limitations, incorporating complex skip patterns and a broad and varying number of portion-size options for extensive food groups. In addition, FFQs administered electronically do not require posterior manual introduction of the collected data [14,23-27]. However, all FFQs (paper and digital format) depend on the long-term memory of the interviewed subject, and they do not take day-to-day intrapersonal variation into account during the period of the study [2,7,14,17-20]. For these reasons, developing new methods that overcome the limitations of FFQs to assess the habitual intake of selected food groups in large-scale epidemiological and intervention studies is well motivated.

The aim of this study was to evaluate the *relative validity* of the current-day dietary recall (current-day recall), a method which is based on a smartphone app called electronic 12-hour dietary recall (e-12HR). Moreover, 4 estimated DRs and a semiquantitative FFQ were used as reference methods to verify comparability of the data with regard to 10 selected food groups among the whole sample and across different strata (sociodemographic characteristics, lifestyle factors, and weight category).

### Previous Research

When researching the habitual consumption of selected food groups, it is essential to be aware of factors that could possibly affect reporting accuracy: gender, age, ethnic group, education level, occupation, employment status, socioeconomic status, diagnosed diseases, a sedentary lifestyle, slimming regimens, smoking, alcohol consumption, weight category, psychological factors, etc [28-31].

This study is an extension of the study previously published in *JMIR mHealth and uHealth* titled *Electronic 12-Hour Dietary Recall (e-12HR): Comparison of a Mobile Phone App for Dietary Intake Assessment With a Food Frequency Questionnaire and Four Dietary Records* [32]. This study, with regard to the previous one, compares e-12HR against an FFQ and 4 DRs in a whole sample of adults (students and employees of the Schools of Medicine or Pharmacy, University of Seville), and in different strata thereof (sociodemographic characteristics, lifestyle factors and weight category), and not only a sample of the university students. It is true that the research team uses, consciously, exactly the same protocol and the same statistical analysis with the idea of making the results comparable. However, in the study at hand, the sample is increased to include new population groups. This has allowed the research team to analyze, on the one hand, if the study objective has been achieved (determining the *relative validity* of current-day recall), which can be extrapolated to a wider amount of the target population, and on the other hand, the influence of certain factors (age, gender, occupation, smoking status, physical activity status, and weight category).

## Methods

### Procedure

The investigation protocol has been published previously elsewhere [32]. In brief, the study was carried out in 2 centers: the Schools of Medicine and Pharmacy at the University of Seville (Andalusia, Spain, South of Europe). Different events were organized to present the project to the students and employees from both faculties. Participant recruitment took place from January 2017 to December 2017. The participants were incorporated in the study progressively during the entire recruitment period in such a way that every day of the week and every season of the year would be represented [33].

Inclusion criteria were as follows: (1) older than 18 years of age, (2) a student or employee of the Schools of Medicine or Pharmacy (University of Seville), and (3) possesses a smartphone with internet access (3G/4G/Wi-Fi) and an Android operating system.

All procedures on human beings were approved by the Research Ethics Committee at the University of Seville.

In the first interview, the participants started by providing informed consent; then, they were assigned a unique alphanumeric code to preserve their anonymity in accordance with current Spanish legislation [34], and they performed the following activities:

1. Each participant filled out an initial questionnaire (on paper), which included the date of the interview and self-reported date of birth, gender, occupation, weight and height measurements, as well as smoking and physical activity status. Body mass index (BMI; kg/m^2^) was estimated from self-reported body weight and height [13,14,35].
2. Each participant downloaded the e-12HR app for their personal smartphone, and a member of the research team personally explained how to use the app with a practical demonstration before written instructions were given to the participants [12,36] to be consulted later if necessary.
3. The same research team member personally gave each participant detailed instructions on how to complete the 4 estimated DRs and how to estimate serving sizes consumed. In addition, an explanatory pamphlet was also given to the participants [10,11].

Written instructions (“how to use the app,” “how to complete the four estimated DRs,” and “how to estimate serving sizes consumed”) are subject to copyright and thus are not included in the manuscript.

**Figure 1:**
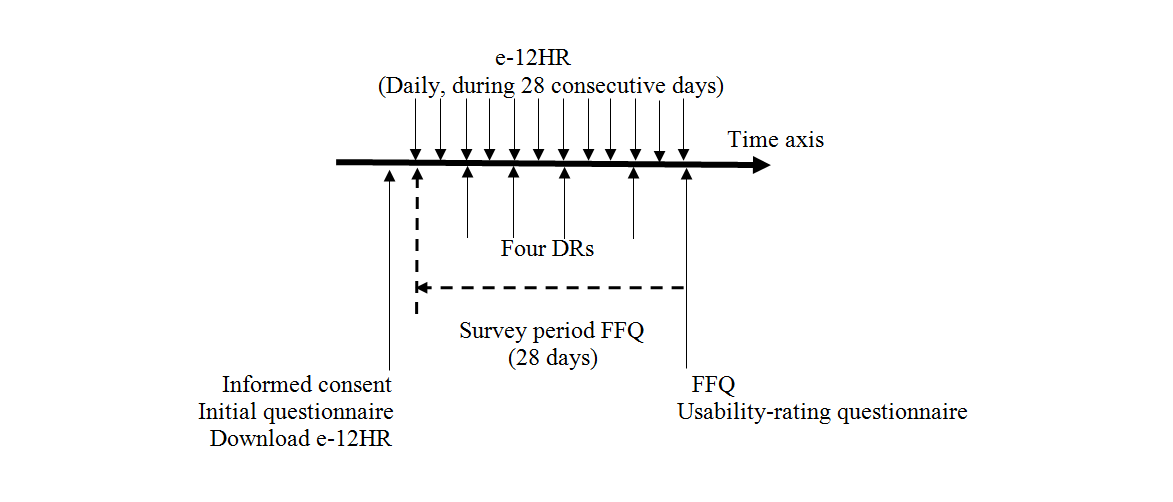
Assessment process using the electronic 12-hour dietary recall (e-12HR) app, 4 dietary records (DRs), food frequency questionnaire (FFQ), and usability rating questionnaire for the e-12HR app.

In the second interview, at the end of the e-12HR app data collection period and at the convenience of each participant, the participant was required to fill out a semiquantitative FFQ. A research team member explained to each participant the process for completing the FFQ. Finally, each participant filled out a usability rating questionnaire [14,28,37] for e-12HR app (Figure 1), which comprised 5 questions about the completion of e-12HR (Multimedia Appendix 1).

### The Electronic 12-Hour Dietary Recall App

The e-12HR app was developed to record daily consumption of a list of 10 food groups: fruit, vegetables, legumes, chicken/turkey, fish, red meat, soft drinks, sweets, prepared foods, and beer (Multimedia Appendix 2). Other food groups such as dairy and derivatives, eggs, nuts, potatoes, pasta, rice, or bread have not been included. In any case, the food groups included can be modified to meet the needs of each study [32]. The list could not be too long to minimize the workload of the participants as well as the research costs [21]. These food groups were selected as they are indicators of health/disease and are considered protective factors (fruit, vegetables, legumes, or fish) or risk factors (soft drinks, commercial baked foods, and precooked meals) for chronic illnesses [1,10,38]. They also provide consumption patterns that range from almost every day for every inhabitant of the population to infrequently for the majority [1].

When the e-12HR app was used the first time, the participants were required to introduce their personally assigned alphanumeric code and the email of the researcher who would receive the data from the app. Participants were instructed to use the app after consuming the last food of the day [12,36]. For each food group, the participant would choose the most appropriate image (or images) from a series of color photographs with 2 to 4 possible options, shown simultaneously [12,36], that illustrated the different serving sizes to assist with selecting the number of standard servings consumed [7,10,12,28,36]. To further assist with estimating serving sizes, each photograph was accompanied by an explanatory text and 3 objects of known/predictable size [39,40] (fiducial markers): a commonly used pencil, pen, and a marker. For example, on the screen of the app, the following would appear: *How many servings of soft drinks have you had today?*, with the *Rations* button and *Next* button. Supposing that the participant had, throughout the day, consumed 2 cans of soft drinks, 1 normal size and another larger size, they would proceed as follows: (1) tap the *Rations* button-a new window opens with different photographs of soft drinks, an *Accept* button, and a *Cancel* button; (2) tap once on the photo corresponding to the normal size; (3) scroll down on the screen; (4) tap once on the photo corresponding to the large size; (5) tap the *Accept* button-the app returns to the previous window; and (6) tap the *Next* button to access the next food group and proceed as before-if an error occurs, the participant can tap the *Cancel* button instead of *Accept*, starting the process over again (Multimedia Appendix 3).

After completing the daily questionnaire with the e-12HR app, the information is automatically saved and sent, via 3G/4G/Wi-Fi, to the e-mail address of the research administrator. Once the questionnaire is completed and sent, the participant cannot change their responses or access the app until the following day.

The consumption record of the selected food groups on the app was performed for 28 consecutive days. The time interval selected is similar to other comparison/validation studies [3,10,13,14,35].

The questionnaire and the size of the rations used in the e-12HR app are based on a semiquantitative FFQ previously validated for the population of Spain [41].

### Dietary Records

During the 28-day period that e-12HR was in use for each participant, 4 estimated DRs (on paper) were scheduled on randomly assigned, nonconsecutive days [9,13]: 3 days during the weekdays and 1 day during the weekend [9-11,13]. The choice between 3 and 7 DRs is normally considered sufficient to evaluate food group intake [42]. Four estimated DRs were chosen instead of weighed DRs for logistical reasons [9,10].

Each participant, during the first interview, received an explanation of how to use the estimated DRs and how to estimate the serving size consumed, through the use of a pamphlet with a series of 2 to 4 color photographs [7,11,12,36] (1 series for each food group). To assist with estimating serving sizes, each photograph was accompanied by an explanatory text and 3 reference objects of known/predictable size [39,40] (fiducial markers). The explanatory text and the fiducial markers were the same as for the e-12HR app.

The DRs used were based on a DR previously validated for another European country (Denmark) [11,43], but structured according to the typical Spanish diet (breakfast, lunch, an afternoon snack, and dinner), and precodified including the same 10 food groups selected for e-12HR. The precoded DR includes 10 rows (1 for each of the food groups selected by the study) and 3 columns for morning, afternoon and evening, and night (Multimedia Appendix 4). This was done to minimize the burden on the participants. The serving sizes were based on a semiquantitative FFQ previously validated for the Spanish population [41].

Participants were told that they must record the consumption data on a separate page for each day [29] and immediately after consuming the food [11,29].

### Food Frequency Questionnaire

The FFQ was a structured, semiquantitative FFQ (on paper) that included the same 10 food groups selected for the e-12HR app and the DRs. A research team member provided participants with an explanatory pamphlet to estimate what was considered a standard serving for each food group. This pamphlet contained a photograph of a standard serving for each food group along with an explanatory text and 3 reference objects of a known/predictable size [39,40] (fiducial markers). The explanatory text and the fiducial markers were the same as for the e-12HR app and the DRs (for a standard serving). The time period considered by the FFQ corresponded to the 28 days of the app. All the participants completed the FFQ within the first week of finishing the e-12HR app with the exception of 4 participants, who completed the FFQ 8 to 14 days later.

The semiquantitative FFQ as well as the standard serving sizes were based on a semiquantitative FFQ previously validated for the Spanish population [41].

### Data Conversion

Using e-12HR, each participant recorded the number of standard serving sizes consumed daily for each food group throughout the 28-day study period. With the 4 estimated DRs, each participant collected the number of standard serving sizes consumed daily for each food group on 4 different days throughout the 28-day monitoring period. On the semiquantitative FFQ, each participant selected the number of standard serving sizes habitually consumed for each food group throughout the 28-day monitoring period (Figure 1).

For each participant, the data from the e-12HR app, the 4 DRs, and the FFQ had to be expressed in the same categories of habitual consumption to make comparisons (6 categories: *less than once a week, once or twice a week, 3-4 times a week, 5-6 times a week, once or twice a day,* and *3 or more times a day*). On the FFQ, these different options for habitual consumption were already available for the participants to choose from, and as such, the FFQ data were not modified. With regard to the e-12HR app, the data needed to be transformed. As an example, 1 participant registered an average daily consumption of 0.76 standard servings of vegetables over 28 days using the app. This average consumption represents 5.32 standard servings per week (0.76×7=5.32), which would be classified as *5 to 6 times a week* [32,44,45]. As for the 4 DRs, the information they contained also needed to be converted [9]. As an example, 1 participant recorded consuming 0, 0.5, and 1 standard pieces of red meat on the DRs during the weekdays and 0.5 standard pieces of red meat on the DR completed at the weekend. This represents an average daily consumption during weekdays of: (0 standard pieces+0.5 standard pieces+1 standard piece)/3 weekdays=0.5 standard pieces per weekday. For weekly consumption, the conversion was as follows: (0.5×5 weekdays)+(0.5×2 weekend days)=2.5+1=3.5 standard pieces, which would then be classified as *3 to 4 times a week*.

To make comparisons, the 3 tools registered the consumption of the same food groups, used the same standard servings as a reference, and the intake record corresponded to the same time period, to avoid possible variations in individual diets during different periods [13,22,28,46].

### Statistical Analysis

The food group intakes estimated by the e-12HR app, the FFQ, and the DRs were categorized into sextiles. For each food group, the consumption category assigned by e-12HR is compared with the category assigned by each of the different reference methods (FFQ and 4 DRs). The association between dietary intake methods (the current-day recall vs the FFQ and vs the 4 DRs) was assessed using Spearman correlation coefficients (SCC) [4]. Cross-classification analysis and kappa statistic index [4] were used as a measure of agreement between the current-day recall and the FFQ/DRs. The proportion of subjects categorized in the same sextile by the different methods (labeled *exact agreement*), in the same or adjacent sextile (labeled exact *agreement + adjacent*), and in opposite sextiles (labeled *extreme disagreement*) was calculated. Kappa statistic index was weighted to take into account the degree of disagreement between the instruments, assigning partial credit to scores using the Stata prerecorded weights [47].

SCC can have a value between -1 and +1; according to Cohen cut-offs, r=±0.5 is considered strong, r=±0.30 is moderate, and r=±0.10 is weak [48]. Weighted kappa statistic index can oscillate between 1 and +1: values of weighted kappa statistic index over 0.80 indicate very good agreement, between 0.80 and 0.61 indicate good agreement, between 0.60 and 0.41 indicate moderate agreement, between 0.40 and 0.21 indicate fair agreement, and <0.20 indicate poor agreement [49]. The comparison criteria considered in this study were as follows: SCC≥0.5 [4,13]; cross-classification percentage in the *exact agreement category* ≥35.0% [13], in the *exact agreement+adjacent category* ≥75.0% [13], and in the *extreme disagreement category* ≤8.0% [14]; and a weighted kappa statistic index ≥0.41 [4].

All statistical tests were 2 sided, and a significance level was considered at *P* value <.05. All data were analyzed using the statistical software STATA version MP 13.1 (Stata Corp LP, College Station, Texas, USA) [47].

It is important to note that the cross-classification analysis and weighted kappa depend on the number of categories used [4]. For example, imagine 2 participants in the study, participants *A* and *B*. Participant *A* presents an average consumption of a specific food group of 3.2 standard servings per week; participant *B* presents an average consumption of the same food group of 5.4 standard servings per week. If the categories considered in the study were 3 categories (Category 1: less than 3 times a week; Category 2: 3-6 times a week; and Category 3: once or more times a day), both participants (*A* and *B*) would be included in category 2. However, if the categories considered in the study are 6 categories (Category 1: less than once a week; Category 2: once or twice a week; Category 3: 3-4 times a week; Category 4: 5-6 times a week; Category 5: once or twice a day; and Category 6: 3 or more times a day), then participant *A* would be included in category 3, whereas participant *B* would be included in category 4.

## Results

### Overview

Of the 217 participants who signed the informed consent, 14 did not complete the study. The results of these individuals were not included in later statistical analysis. Information on the number of days completed with e-12HR can be found in Table 1. To highlight, 58.1% (118/203) of the participants completed the task every day (28 days of monitoring), 10.3% (21/203) completed the task 27 days, and 11.8% (24/203) completed the app 26 days.

The average age of the participants was 32 years. Moreover, 59.6% (121/203) were ≥25 years old; 56.7% (115/203) were women. In addition, 57.1% (116/203) were employees and 42.9% (87/203) were students. A majority (83.7% [170/203]) of the participants were nonsmokers. Two-thirds (66.5% [135/203]) of the respondents performed 150 min or more of moderate-intensity physical activities per week [50]. The mean BMI was 24.2 kg/m^2^, with 4.9% (10/203) of the participants in the underweight range (BMI<18.5), 61.1% (124/203) in the healthy weight range (BMI: 18.5-24.9), 25.1% (51/203) being overweight (BMI: 25.0-29.9), and 8.9% (18/203) obese (BMI>30.0) [51] (Table 1). No statistically significant differences in the variables studied were found among the participants who completed the study and those who did not.

**Table 1:** Characteristics of study participants.

Characteristics		Statistics, n (%)	Mean (SD)	95% CI
Participants who completed the study		203 (100)	—^a^	—
**Number of days completed the app**				
	28 days	118 (58.1)	—	—
	27 days	21 (10.3)	—	—
	26 days	24 (11.8)	—	—
	25 days	12 (5.9)	—	—
	24 days	11 (5.4)	—	—
	23 days	6 (3.0)	—	—
	22 days	7 (3.4)	—	—
	21 days	4 (2.0)	—	—
**Age (years)**		—	32.0 (11.4)	—
	<25	82 (40.4)	—	33.6-47.2
	≥25	121 (59.6)	—	52.8-66.4
**Gender**				
	Females	115 (56.7)	—	49.8-63.5
	Males	88 (43.3)	—	36.5-50.2
**Occupation**				
	Students	87 (42.9)	—	36.0-49.7
	Employees	116 (57.1)	—	50.3-64.0
**Smoking status**				
	No	170 (83.7)	—	78.6-88.9
	Yes	33 (16.3)	—	11.1-21.4
**Physical activity status (minutes/week)**				
	≥150	135 (66.5)	—	60.0-73.1
	<150	68 (33.5)	—	26.9-40.0
**Body mass index (kg/m^2^>)**		—	24.2 (4.1)	—
	<25	134 (66.0)	—	59.4-72.6
	≥25	69 (34.0)	—	27.4-40.6

^a^—: not applicable.

### The Electronic 12-Hour Dietary Recall App Versus the Food Frequency Questionnaire

For all the food groups, for all participants, and for all strata, the average SCC was 0.67 (by strata, from 0.62 [smokers] to 0.70 [student]; Tables 2 and 3). Cross-classification analysis showed that the average percentage of individuals classified in the *exact agreement* category was 50.7% (by strata, from 47.0% [males] to 53.6% [females]); *exact agreement+adjacent* was 90.2% (by strata, from 88.6% [BMI≥25] to 91.8% [students]); and no participants (0%) were classified in the *extreme disagreement* category (Tables 4 and 5; see Multimedia Appendix 5 for full details). The average weighted kappa was .49 (by strata, from 0.44 [smokers] to 0.51 [students]; Tables 6 and 7).

**Table 2:** Spearman correlation coefficients derived from the electronic 12-hour dietary recall app versus the food frequency questionnaire for categories of food group consumption and strata: age (years): <25 and ≥25; gender: females and males; and occupation: students and employees. Comparison of the electronic 12-hour dietary recall app versus food frequency questionnaire. Spearman correlation coefficients (95% CI).

Food groups	All^a^	Age (years)		Gender		Occupation	
		<25	≥25	Females	Males	Students	Employees
Fruit	0.80 (0.75-0.84)	0.82 (0.73-0.88)	0.79 (0.71-0.85)	0.76 (0.67-0.83)	0.85 (0.78-0.90)	0.84 (0.76-0.89)	0.77 (0.68-0.83)
Vegetables	0.70 (0.62-0.76)	0.77 (0.66-0.85)	0.64 (0.52-0.74)	0.70 (0.60-0.78)	0.69 (0.56-0.78)	0.80 (0.71-0.86)	0.61 (0.48-0.71)
Legumes	0.48 (0.37-0.58)	0.47 (0.29-0.63)	0.51 (0.37-0.63)	0.51 (0.36-0.63)	0.46 (0.27-0.61)	0.50 (0.32-0.64)	0.49 (0.34-0.62)
Chicken/Turkey	0.58 (0.48-0.67)	0.49 (0.31-0.64)	0.63 (0.51-0.73)	0.58 (0.45-0.69)	0.60 (0.45-0.72)	0.53 (0.36-0.66)	0.62 (0.49-0.72)
Fish	0.53 (0.42-0.62)	0.62 (0.47-0.74)	0.48 (0.33-0.60)	0.52 (0.37-0.64)	0.56 (0.40-0.69)	0.65 (0.51-0.76)	0.44 (0.28-0.58)
Red meat	0.63 (0.53-0.70)	0.71 (0.58-0.80)	0.58 (0.45-0.69)	0.65 (0.53-0.75)	0.54 (0.37-0.67)	0.69 (0.56-0.79)	0.58 (0.44-0.69)
Soft drinks	0.83 (0.78-0.87)	0.79 (0.69-0.86)	0.85 (0.79-0.89)	0.82 (0.75-0.87)	0.84 (0.77-0.89)	0.81 (0.73-0.87)	0.85 (0.78-0.89)
Sweets	0.72 (0.64-0.78)	0.72 (0.59-0.81)	0.71 (0.61-0.79)	0.71 (0.61-0.79)	0.74 (0.63-0.82)	0.71 (0.59-0.80)	0.71 (0.60-0.79)
Prepared foods	0.59 (0.49-0.67)	0.57 (0.40-0.70)	0.59 (0.46-0.69)	0.57 (0.44-0.69)	0.61 (0.46-0.73)	0.61 (0.46-0.73)	0.55 (0.41-0.66)
Beer	0.88 (0.85-0.91)	0.86 (0.78-0.90)	0.88 (0.84-0.92)	0.87 (0.82-0.91)	0.88 (0.81-0.92)	0.86 (0.80-0.91)	0.89 (0.84-0.92)
Average	0.67 (—^b^)	0.68 (—)	0.67 (—)	0.67 (—)	0.68 (—)	0.70 (—)	0.65 (—)

^a^*P*<.001 for all data.

^b^—: not applicable.

**Table 3:** Spearman correlation coefficients derived from the electronic 12-hour dietary recall app versus the food frequency questionnaire for categories of food group consumption and strata: smoking: no and yes; physical activity (minutes/week): ≥150 and <150; and body mass index (kg/m^2^): <25 and ≥25. Comparison of the electronic 12-hour dietary recall app versus food frequency questionnaire. Spearman correlation coefficients (95% CI).

Food groups	All^a^	Smoking		Physical activity (minutes/week)		Body mass index (kg/m^2^)	
		No	Yes	≥150	<150	<25	≥25
Fruit	0.80 (0.75-0.84)	0.80 (0.74-0.85)	0.81 (0.64-0.90)	0.80 (0.72-0.85)	0.81 (0.71-0.88)	0.78 (0.70-0.84)	0.85 (0.77-0.91)
Vegetables	0.70 (0.62-0.76)	0.73 (0.65-0.79)	0.53^b^ (0.22-0.74)	0.70 (0.60-0.77)	0.64 (0.48-0.76)	0.69 (0.59-0.77)	0.75 (0.62-0.84)
Legumes	0.48 (0.37-0.58)	0.49 (0.37-0.60)	0.41^b^ (0.07-0.66)	0.46 (0.32-0.59)	0.52 (0.33-0.68)	0.47 (0.33-0.59)	0.51 (0.31-0.66)
Chicken/Turkey	0.58 (0.48-0.67)	0.63 (0.52-0.71)	0.38^b^ (0.04-0.64)	0.57 (0.44-0.67)	0.61 (0.44-0.74)	0.61 (0.49-0.71)	0.54 (0.34-0.69)
Fish	0.53 (0.42-0.62)	0.52 (0.40-0.62)	0.59 (0.31-0.78)	0.52 (0.39-0.64)	0.51 (0.31-0.67)	0.58 (0.45-0.68)	0.42 (0.21-0.60)
Red meat	0.63 (0.53-0.70)	0.62 (0.52-0.71)	0.64 (0.38-0.81)	0.64 (0.53-0.73)	0.60 (0.42-0.73)	0.62 (0.50-0.71)	0.64 (0.47-0.76)
Soft drinks	0.83 (0.78-0.87)	0.83 (0.77-0.87)	0.85 (0.72-0.92)	0.84 (0.79-0.89)	0.80 (0.70-0.87)	0.82 (0.76-0.87)	0.83 (0.73-0.89)
Sweets	0.72 (0.64-0.78)	0.72 (0.64-0.79)	0.67 (0.42-0.82)	0.71 (0.62-0.79)	0.75 (0.62-0.84)	0.71 (0.61-0.78)	0.74 (0.61-0.83)
Prepared foods	0.59 (0.49-0.67)	0.62 (0.52-0.71)	0.44^b^ (0.12-0.68)	0.58 (0.45-0.68)	0.59 (0.41-0.72)	0.58 (0.45-0.68)	0.61 (0.43-0.74)
Beer	0.88 (0.85-0.91)	0.87 (0.83-0.91)	0.88 (0.77-0.94)	0.91 (0.88-0.94)	0.82 (0.73-0.89)	0.91 (0.87-0.93)	0.85 (0.76-0.90)
Average	0.67 (—^c^)	0.68 (—)	0.62 (—)	0.67 (—)	0.67 (—)	0.68 (—)	0.67 (—)

^a^*P*<.001 for all data, except:

^b^*P*<.05.

^c^—: not applicable.

**Table 4:** Cross-classification analysis derived from the electronic 12-hour dietary recall app versus the food frequency questionnaire for categories of food group consumption and strata: age (years): <25 and ≥25; gender: females and males; and occupation: students and employees. Comparison of the electronic 12-hour dietary recall app versus food frequency questionnaire.

Agreement	All^a^	Age (years)		Gender		Occupation	
		<25	≥25	Females	Males	Students	Employees
Exact agreement^b^ (%)	50.7	50.9	50.7	53.6	47.0	51.5	50.2
Exact agreement + adjacent^c^ (%)	90.2	90.7	90.0	90.9	89.5	91.8	89.2
Extreme disagreement^d^ (%)	0.0	0.0	0.0	0.0	0.0	0.0	0.0

^a^Data presented are mean agreement for 10 different food groups: fruit, vegetables, legumes, chicken/turkey, fish, red meat, soft drinks, sweets, prepared foods, and beer.

^b^Exact agreement: cases cross-classified into the same category.

^c^Exact agreement + adjacent: cases cross-classified into the same or adjacent category.

^d^Extreme disagreement: cases cross-classified into extreme categories.

**Table 5:** Cross-classification analysis derived from the electronic 12-hour dietary recall app versus the food frequency questionnaire for categories of food group consumption and strata: smoking: no and yes; physical activity (minutes/week): ≥150 and <150; and body mass index (kg/m^2^): <25 and ≥25. Comparison of the electronic 12-hour dietary recall app versus the food frequency questionnaire.

Agreement	All^a^	Smoking		Physical activity (minutes/week)		Body mass index (kg/m^2^)	
		No	Yes	≥150	<150	<25	≥25
Exact agreement^b^ (%)	50.7	50.9	49.7	51.4	49.4	51.5	49.6
Exact agreement + adjacent^c^ (%)	90.2	90.4	90.0	90.4	90.0	91.3	88.6
Extreme disagreement^d^ (%)	0.0	0.0	0.0	0.0	0.0	0.0	0.0

^a^Data presented are mean agreement for 10 different food groups: fruit, vegetables, legumes, chicken/turkey, fish, red meat, soft drinks, sweets, prepared foods, and beer.

^b^Exact agreement: cases cross-classified into the same category.

^c^Exact agreement + adjacent: cases cross-classified into the same or adjacent category.

^d^Extreme disagreement: cases cross-classified into extreme categories.

**Table 6:** Weighted kappa derived from the electronic 12-hour dietary recall app versus the food frequency questionnaire for categories of food group consumption and strata: age (years): <25 and ≥25; gender: females and males; and occupation: students and employees. Comparison of the electronic 12-hour dietary recall app versus the food frequency questionnaire.

Food groups	All^a^	Age (years)		Gender		Occupation	
		<25	≥25	Females	Males	Students	Employees
Fruit	0.65	0.64	0.64	0.63	0.67	0.66	0.63
Vegetables	0.54	0.59	0.48	0.59	0.48	0.61	0.46
Legumes	0.37	0.35	0.38	0.38	0.34	0.38	0.37
Chicken/Turkey	0.40	0.32	0.44	0.34	0.46	0.34	0.44
Fish	0.30	0.37	0.26	0.29	0.32	0.41	0.22
Red meat	0.46	0.55	0.38	0.48	0.37	0.54	0.39
Soft drinks	0.59	0.52	0.62	0.60	0.58	0.59	0.60
Sweets	0.49	0.48	0.48	0.51	0.45	0.48	0.47
Prepared foods	0.40	0.38	0.40	0.40	0.40	0.41	0.36
Beer	0.68	0.67	0.66	0.64	0.67	0.66	0.67
Average	0.49	0.49	0.48	0.49	0.48	0.51	0.46

^a^*P*<.001 for all data.

### The Electronic 12-Hour Dietary Recall App Versus the 4 Dietary Records

For all the food groups, for all participants, and for all strata, the average SCC was 0.65 (by strata, from 0.57 [smokers] to 0.67 [males]; Tables 8 and 9). Cross-classification analysis showed that the average percentage of individuals classified in the *exact agreement* category was 50.0% (by strata, from 47.0% [males] to 52.3% [females]); *exact agreement+adjacent* was 88.2% (by strata, from 86.1% [males] to 89.8% [females]); and no participants (0%) were classified in the *extreme disagreement* category (Tables 10 and 11; see Multimedia Appendix 5 for full details). The average weighted kappa was .50 (by strata, from 0.44 [smokers] to 0.50 [≥25 years, males, employees, non-smokers, ≥150 min/week, and BMI<25]; Tables 12 and 13).

**Table 7:** Weighted kappa derived from the electronic 12-hour dietary recall app versus the food frequency questionnaire for categories of food group consumption and strata: smoking: no and yes; physical activity (minutes/week): ≥150 and <150; and body mass index (kg/m^2^): <25 and ≥25. Comparison of the electronic 12-hour dietary recall app versus the food frequency questionnaire.

Food groups	All^a^	Smoking		Physical activity (minutes/week)		Body mass index (kg/m^2^)	
		No	Yes	≥150	<150	<25	≥25
Fruit	0.65	0.65	0.65	0.63	0.67	0.63	0.68
Vegetables	0.54	0.56	0.43^b^	0.56	0.48	0.54	0.55
Legumes	0.37	0.37	0.33^b^	0.37	0.35	0.35	0.40
Chicken/Turkey	0.40	0.43	0.26^b^	0.39	0.41	0.45	0.31
Fish	0.30	0.30	0.34^b^	0.30	0.30	0.34	0.23^b^
Red meat	0.46	0.46	0.45	0.44	0.49	0.46	0.43
Soft drinks	0.59	0.59	0.60	0.62	0.55	0.60	0.55
Sweets	0.49	0.49	0.44	0.51	0.45	0.48	0.49
Prepared foods	0.40	0.44	0.22^b^	0.41	0.37	0.39	0.44
Beer	0.68	0.67	0.66	0.71	0.61	0.70	0.63
Average	0.49	0.50	0.44	0.49	0.47	0.49	0.47

^a^*P*<.001 for all data, except:

^b^*P*<.05.

**Table 8:** Spearman correlation coefficients derived from the electronic 12-hour dietary recall app versus the 4 dietary records for categories of food group consumption and strata: age (years): <25 and ≥25; gender: females and males; and occupation: students and employees. Comparison of the electronic 12-hour dietary recall app versus dietary records. Spearman correlation coefficients (95% CI).

Food groups	All^a^	Age (years)		Gender		Occupation	
		<25	≥25	Females	Males	Students	Employees
Fruit	0.78 (0.73-0.83)	0.82 (0.74-0.88)	0.74 (0.65-0.81)	0.80 (0.72-0.86)	0.76 (0.65-0.83)	0.83 (0.75-0.88)	0.74 (0.64-0.81)
Vegetables	0.72 (0.65-0.78)	0.74 (0.63-0.83)	0.68 (0.56-0.76)	0.70 (0.60-0.78)	0.75 (0.64-0.83)	0.72 (0.60-0.81)	0.69 (0.58-0.77)
Legumes	0.40 (0.28-0.51)	0.40 (0.20-0.56)	0.43 (0.27-0.56)	0.34 (0.17-0.50)	0.47 (0.29-0.62)	0.46 (0.27-0.61)	0.39 (0.22-0.53)
Chicken/Turkey	0.63 (0.54-0.71)	0.59 (0.42-0.71)	0.67 (0.56-0.76)	0.63 (0.51-0.73)	0.60 (0.44-0.72)	0.60 (0.45-0.72)	0.66 (0.54-0.75)
Fish	0.52 (0.41-0.61)	0.53 (0.35-0.67)	0.49 (0.34-0.62)	0.50 (0.35-0.63)	0.55 (0.38-0.68)	0.47 (0.28-0.62)	0.51 (0.36-0.63)
Red meat	0.52 (0.42-0.62)	0.50 (0.32-0.65)	0.54 (0.40-0.66)	0.45 (0.29-0.59)	0.55 (0.38-0.68)	0.50 (0.32-0.64)	0.54(0.39-0.65)
Soft drinks	0.77 (0.71-0.82)	0.70 (0.57-0.79)	0.82 (0.75-0.87)	0.73 (0.64-0.81)	0.82 (0.74-0.88)	0.72 (0.60-0.81)	0.81 (0.74-0.86)
Sweets	0.72 (0.64-0.78)	0.78 (0.68-0.85)	0.68 (0.57-0.77)	0.71 (0.61-0.79)	0.74 (0.63-0.82)	0.74 (0.63-0.82)	0.70 (0.60-0.79)
Prepared foods	0.63 (0.53-0.70)	0.57 (0.40-0.70)	0.66 (0.55-0.75)	0.62 (0.49-0.72)	0.67 (0.53-0.77)	0.60 (0.45-0.72)	0.63 (0.51-0.73)
Beer	0.81 (0.75-0.85)	0.71 (0.59-0.80)	0.84 (0.77-0.88)	0.83 (0.76-0.88)	0.77 (0.67-0.84)	0.70 (0.58-0.80)	0.85 (0.78-0.89)
Average	0.65 (—^b^)	0.63 (—)	0.65 (—)	0.63 (—)	0.67 (—)	0.63 (—)	0.65 (—)

^a^*P*<.001 for all data.

^b^—: not applicable.

**Table 9:** Spearman correlation coefficients derived from the electronic 12-hour dietary recall app versus the 4 dietary records for categories of food group consumption and strata: smoking: no and yes; physical activity (minutes/week): ≥150 and <150; and body mass index (kg/m^2^): <25 and ≥25. Comparison of the electronic 12-hour dietary recall app versus dietary records. Spearman correlation coefficients (95% CI).

Food groups	All^a^	Smoking		Physical activity (minutes/week)		Body mass index (kg/m^2^)	
		No	Yes	≥150	<150	<25	≥25
Fruit	0.78 (0.73-0.83)	0.79 (0.72-0.84)	0.72 (0.50-0.85)	0.76 (0.68-0.83)	0.82 (0.72-0.88)	0.75 (0.67-0.82)	0.82 (0.73-0.89)
Vegetables	0.72 (0.65-0.78)	0.74 (0.66-0.80)	0.61 (0.34-0.79)	0.72 (0.63-0.79)	0.69 (0.54-0.80)	0.70 (0.60-0.77)	0.78 (0.66-0.86)
Legumes	0.40 (0.28-0.51)	0.42 (0.29-0.53)	0.29^c^ (0.00-0.57)	0.40 (0.25-0.54)	0.39^b^ (0.17-0.58)	0.38 (0.23-0.52)	0.43 (0.21-0.60)
Chicken/Turkey	0.63 (0.54-0.71)	0.64 (0.54-0.72)	0.54^b^ (0.25-0.75)	0.60 (0.48-0.70)	0.70 (0.55-0.80)	0.63 (0.52-0.72)	0.63 (0.46-0.76)
Fish	0.52 (0.41-0.61)	0.51 (0.38-0.61)	0.57^b^ (0.28-0.76)	0.54 (0.41-0.65)	0.46 (0.25-0.63)	0.63 (0.51-0.72)	0.33^b^ (0.10-0.53)
Red meat	0.52 (0.42-0.62)	0.55 (0.44-0.65)	0.31^d^ (0.00-0.59)	0.59 (0.47-0.69)	0.39^b^ (0.17-0.58)	0.52 (0.39-0.64)	0.57 (0.38-0.71)
Soft drinks	0.77 (0.71-0.82)	0.77 (0.71-0.83)	0.75 (0.54-0.87)	0.79 (0.72-0.85)	0.73 (0.59-0.82)	0.77 (0.69-0.83)	0.75 (0.62-0.84)
Sweets	0.72 (0.64-0.78)	0.72 (0.64-0.79)	0.54^b^ (0.24-0.74)	0.72 (0.63-0.79)	0.72 (0.59-0.82)	0.73 (0.64-0.80)	0.70 (0.56-0.80)
Prepared foods	0.63 (0.53-0.70)	0.63 (0.53-0.71)	0.64 (0.38-0.81)	0.61 (0.49-0.70)	0.67 (0.51-0.78)	0.59 (0.47-0.69)	0.68 (0.53-0.79)
Beer	0.81 (0.75-0.85)	0.81 (0.74-0.85)	0.78 (0.59-0.89)	0.80 (0.73-0.85)	0.82 (0.73-0.89)	0.81 (0.74-0.86)	0.82 (0.72-0.88)
Average	0.65 (—^e^)	0.66 (—)	0.57 (—)	0.65 (—)	0.64 (—)	0.65 (—)	0.65 (—)

^a^*P*<.001 for all data, except:

^b^*P*<.05.

^c^*P*=.106.

^d^*P*=.083.

^e^—: not applicable.

**Table 10:** Cross-classification analysis derived from the electronic 12-hour dietary recall app versus the 4 dietary records for categories of food group consumption and strata: age (years): <25 and ≥25; gender: females and males; and occupation: students and employees. Comparison of the electronic 12-hour dietary recall app versus dietary records.

Agreement	All^a^	Age (years)		Gender		Occupation	
		<25	≥25	Females	Males	Students	Employees
Exact agreement^b^ (%)	50.0	47.7	51.6	52.3	47.0	47.1	52.2
Exact agreement + adjacent^c^ (%)	88.2	89.3	87.5	89.8	86.1	89.2	87.5
Extreme disagreement^d^ (%)	0.0	0.0	0.0	0.0	0.0	0.0	0.0

^a^Data presented are mean agreement for 10 different food groups: fruit, vegetables, legumes, chicken/turkey, fish, red meat, soft drinks, sweets, prepared foods, and beer.

^b^Exact agreement: cases cross-classified into the same category.

^c^Exact agreement + adjacent: cases cross-classified into the same or adjacent category.

^d^Extreme disagreement: cases cross-classified into extreme categories.

**Table 11:** Cross-classification analysis derived from the electronic 12-hour dietary recall app versus the 4 dietary records for categories of food group consumption and strata: smoking: no and yes; physical activity (minutes/week): ≥150 and <150; and body mass index (kg/m^2^): <25 and ≥25. Comparison of the electronic 12-hour dietary recall app versus dietary records.

Agreement	All^a^	Smoking		Physical activity (minutes/week)		Body mass index (kg/m^2^)	
		No	Yes	≥150	<150	<25	≥25
Exact agreement^b^ (%)	50.0	50.5	47.3	51.0	48.1	51.0	48.0
Exact agreement + adjacent^c^ (%)	88.2	88.1	89.1	88.1	88.4	89.0	86.8
Extreme disagreement^d^ (%)	0.0	0.0	0.0	0.0	0.0	0.0	0.0

^a^Data presented are mean agreement for 10 different food groups: fruit, vegetables, legumes, chicken/turkey, fish, red meat, soft drinks, sweets, prepared foods, and beer.

^b^Exact agreement: cases cross-classified into the same category.

^c^Exact agreement + adjacent: cases cross-classified into the same or adjacent category.

^d^Extreme disagreement: cases cross-classified into extreme categories.

**Table 12:** Weighted kappa derived from the electronic 12-hour dietary recall app versus the 4 dietary records for categories of food group consumption and strata: age (years): <25 and ≥25; gender: females and males; and occupation: students and employees. Comparison of the electronic 12-hour dietary recall app versus dietary records.

Food groups	All^a^	Age (years)		Gender		Occupation	
		<25	≥25	Females	Males	Students	Employees
Fruit	0.67	0.73	0.61	0.69	0.64	0.72	0.61
Vegetables	0.56	0.54	0.53	0.56	0.55	0.51	0.55
Legumes	0.29	0.28^b^	0.30	0.21^b^	0.38	0.34	0.26
Chicken/Turkey	0.43	0.36	0.47	0.42	0.41	0.36	0.47
Fish	0.31	0.29	0.29	0.29	0.33	0.26	0.31
Red meat	0.34	0.34	0.35	0.31	0.32	0.34	0.34
Soft drinks	0.64	0.58	0.67	0.64	0.63	0.60	0.66
Sweets	0.54	0.52	0.56	0.55	0.53	0.49	0.58
Prepared foods	0.47	0.41	0.50	0.43	0.52	0.44	0.47
Beer	0.72	0.64	0.74	0.77	0.65	0.63	0.76
Average	0.50	0.47	0.50	0.49	0.50	0.47	0.50

^a^*P*<.001 for all data, except:

^b^*P*<.05.

### Usability Rating Questionnaire for the Electronic 12-Hour Dietary Recall App

The responses of the participants to the usability-rating questionnaire are shown in Tables 14 and 15.

**Table 13:** Weighted kappa derived from the electronic 12-hour dietary recall app versus the 4 dietary records for categories of food group consumption and strata: smoking: no and yes; physical activity (minutes/week): ≥150 and <150; and body mass index (kg/m^2^): <25 and ≥25. Comparison of the electronic 12-hour dietary recall app versus dietary records.

Food groups	All^a^	Smoking		Physical activity (minutes/week)		Body mass index (kg/m^2^)	
		No	Yes	≥150	<150	<25	≥25
Fruit	0.67	0.68	0.60	0.63	0.74	0.66	0.68
Vegetables	0.56	0.58	0.46	0.59	0.48	0.53	0.61
Legumes	0.29	0.31	0.18^c^	0.32	0.22^b^	0.26	0.34
Chicken/Turkey	0.43	0.43	0.42^b^	0.42	0.43	0.45	0.39
Fish	0.31	0.30	0.34^b^	0.32	0.25^b^	0.39	0.16^b^
Red meat	0.34	0.36	0.19^b^	0.39	0.24^b^	0.35	0.33
Soft drinks	0.64	0.63	0.70	0.64	0.64	0.66	0.58
Sweets	0.54	0.54	0.50	0.54	0.54	0.55	0.54
Prepared foods	0.47	0.48	0.42	0.47	0.47	0.43	0.55
Beer	0.72	0.74	0.63	0.72	0.73	0.71	0.74
Average	0.50	0.50	0.44	0.50	0.47	0.50	0.49

^a^*P*<.001 for all data, except:

^b^*P*<.05

^c^*P*=.0624.

**Table 14:** Responses of the study participants to the usability rating questionnaire for the electronic 12-hour dietary recall app (part 1).

Options	Questions, n (%)			
	Easy to complete	Too time consuming	Interesting to complete	I would be willing to complete again
Strongly agree	139 (68.5)	3 (1.5)	48 (23.6)	57 (28.1)
Agree	62 (30.5)	4 (2.0)	111 (54.7)	104 (51.2)
Neither agree nor disagree	2 (1.0)	4 (2.0)	39 (19.2)	39 (19.2)
Disagree	0 (0.0)	82 (40.4)	5 (2.5)	3 (1.5)
Strongly disagree	0 (0.0)	110 (54.2)	0 (0.0)	0 (0.0)

**Table 15:** Responses of the study participants to the usability rating questionnaire for the electronic 12-hour dietary recall app (part 2).

Options	Question, n (%)
	Time to complete the app
<1 min/day	23 (11.3)
Approximately 1 min/day	54 (26.6)
Approximately 2 min/day	63 (31.0)
Approximately 3 min/day	41 (20.2)
Approximately 4 min/day	16 (7.9)
5 min/day or more	6 (3.0)

## Discussion

### Overview

The current-day recall has been designed to categorize participants according to habitual intake of selected food groups. Notwithstanding, this method is not intended to determine the total amount of foods consumed by an individual nor the exact quantity consumed for specific food groups or nutrients. This method is basically a modified 24-hour recall focused on a series of 10 food groups and completed at the end of every day during 28 consecutive days [32,44,45]. In this study, the current-day recall, based on the e-12HR app, has been compared with 2 different reference models, one long term (FFQ) and the other short-term (4 DRs), in the whole sample of adults and in different strata thereof (sociodemographic characteristics, lifestyle factors, and weight category).

Even though 2 different reference methods were used for e-12HR, the high degree of association and agreement between the data collected when comparing the different methods does not indicate that the current-day recall is exact, as there is no true measurement of dietary intake [2,8,32,52].

### Principal Findings: The Electronic 12-Hour Recall App Versus the Food Frequency Questionnaire and the 4 Dietary Records

For each of the 10 food groups considered in this study, a comparison was made using e-12HR versus FFQ as well as e-12HR versus DRs. In both comparisons, 5 criteria were considered to compare the different methods: SCC; cross-classification percentage in the exact agreement category, in the exact agreement+adjacent category, and in the extreme disagreement category; and weighted kappa. Apart from this, and in the comparisons, the complete sample and the 12 individual strata were compared. This generated 130 statistical indicators for each of the food groups. For example, for fruit, a statistical indicator was obtained for each of the comparison criteria (5 comparison criteria), for the complete sample and the different strata (13 strata), for e-12HR versus FFQ comparison (5×13=65 indicators), and finally for e-12HR versus DRs (5×13=65 indicators). The 130 statistical indicators obtained for each food group fulfilled the comparison criteria (see the *Statistical Analysis* section) for fruit, vegetables, soft drinks, sweets, and beer. For the rest of the food groups, of the 130 statistical indicators obtained for each, the following cases did not fulfill the comparison criteria: legumes, 36.1% (47/130); chicken/turkey, 13.1% (17/130); fish, 30.0% (39/130); red meat, 16.1% (21/130); and prepared foods, 7.7% (10/130).

Regarding the SCC, in all of these cases, the agreement between methods was moderate (r=±0.30), except in e-12HR versus RDs, for legumes, and yes smoking strata (0.29). Regarding the cross-classification percentage in the exact agreement category, in all cases the percentage of agreement between the methods was at least 31%, except for the e-12HR app versus FFQ for chicken/turkey and yes smoking strata (27.3%), as well as e-12HR versus DRs for fish and ≥25 years strata (24.6%). Regarding the weighted kappa, in all cases the agreement between the methods was fair (weighted kappa statistic index between 0.40 and 0.21), except in e-12HR versus RDs, for legumes and the yes smoking strata (0.18); for fish, ≥25 kg/m^2^ strata (0.16); and for red meat, yes smoking strata (0.19; see Multimedia Appendix 6).

Evaluating the true validity of a method requires measuring, with a high degree of accuracy, the habitual diet of free-living individuals during a prolonged period, which is not feasible [4]. As a result, the researchers of this study have evaluated the relative validity of e-12HR by comparing it with 2 alternative methods of dietary assessment (FFQ and DRs), with their own limitations (there is no perfect measure of dietary intake, which implies that validation studies are not possible) [2,3,6,16-19]. Thus, validation studies never compare an operational method with absolute truth. To do so, the lesser degree of agreement between e-12HR and the reference methods for some food groups (especially legumes and fish) does not imply that e-12HR is a bad categorization method for habitual dietary intake for these food groups. The current-day recall is a method that depends only on short-term memory (e-12HR app is completed at the end of each day); it takes day-to-day intrapersonal variation into account during the period of the study (the app is completed daily). At the same time, the FFQ compiles information at the end of the study period, DR only on 4 of the 28 days of the study period. With regard to the FFQs, we must take into account the fact that the recollection of past consumption of foods can be influenced by more recent food consumption [2,6,17,18]. Regarding the DRs, short-term methods are generally unrepresentative of habitual intake if only one or a few days are assessed [2]. The different characteristics of e-12HR, the FFQ, and DRs can contribute to assigning different categories of habitual consumption depending on the method, especially for those food groups that are consumed infrequently, such as, legumes, and fish. In any case, the research team will develop future studies to explore the reasons for the disagreement between the methods for these 5 food groups.

The majority of the published research reports associations between the methods, measured by correlation coefficients, although agreement is the most appropriate comparison for validation studies [8]. As previously mentioned in the *Statistical Analysis* subsection, the different categorization of individuals according to the number of categories considered would affect the cross-classification analysis and weighted kappa. With regard to the cross-classification analysis, the dependence on the number of categories considered is reduced as a result of the comparison considered by Forster et al [13] and Fallaize et al [14]; these studies used 4 categories. However, the weighted kappa suffered, especially due to the comparison criterion considered by Masson et al [4] for weighted kappa being defined for 3 categories instead of the 6 considered here. The 6 original categories could have been reorganized into 3 [4,8,44], 4 [13,14], or 5 [30,53,54], as other authors have done. However, this research team preferred to maintain 6 categories for the statistical analysis [32,45] as a greater number of categories of habitual consumption provides compact information on the ability of the methods to assign individuals according to the distribution of dietary intake [30]. In any case, the values observed indicate high correlation and good agreement between the e-12HR app and the 2 reference methods, in the whole sample and in all strata considered: age group (<25 years old and ≥25 years old), gender (female and male), occupation (student and employee), smoking status (no and yes), physical activity status (≥150 min/week and <150 min/week), and BMI (<25 kg/m^2^ and ≥25 kg/m^2^).

e-12HR presents interesting characteristics for both participants and investigators. For participants, the app is easy, brief, and interesting to complete (according to the usability-rating questionnaire). For investigators, with the e-12HR app, data collection is performed digitally, eliminating the need for investigators to later introduce the data manually; it is a self-reporting tool, not requiring interviewers; and overall research costs are greatly reduced. Notwithstanding, current-day recall presents some weaknesses when determining the category of habitual consumption; although the method only depends on short-term memory, it still depends on the memory of the participant (as e-12HR is not completed immediately after each meal, rather at the end of each day), and the number of different options for servings consumed is limited (with color photographs that represent 2 to 4 possible options). Regarding the use of photographs, when comparing e-12HR with the FFQ, the values obtained are similar to those from a previous study that used only 1 reference method (a semiquantitative FFQ) and the older version of the app (which did not use photographs to facilitate estimation of the servings consumed) [45]. As such, the research team would like to mention that the introduction of photographs in the newer version of the app has not translated into better correlation or agreement data between these 2 methods.

The 3 methods for determining diet refer to the same tracking period to avoid possible variations in the intake of different foods over time [13,22,28,46]. This is especially likely among the university students who make up the sample of this study. Reasons being that dietary intake is variable from day to day, sporadic changes in food intake are common (skipping meals, snacking, school events interfering with meal times), and dining out is more frequent than in the general population [44]. All these reasons could have led to an underestimation of the correlation and agreement between the different methods that were compared. In contrast, using the same period of time could overestimate the correlation and agreement between the different methods compared. There are no bibliographic references from other authors that allow us to evaluate this overestimation of such a new method as current-day recall. In the comparison of e-12HR versus the FFQ, the app was completed daily over 28 consecutive days, and the FFQ was completed after the end of period of app use. It is unlikely that the participant would be able to remember the information collected in the app during the 28-day period and that this reminiscence facilitates completing the FFQ, and overestimating the correlation and agreement between both methods. In the comparison of e-12HR versus DRs, the app is completed during 28 days, and on 4 of them, a DR is completed. On the days on which the participants complete both methods, remembering the answers to the DR will favor completion of the app; however, this only occurs during 4 of the 28 days of the study period, and as such, overestimation of the correlation and agreement of both methods is unlikely to be significant.

#### Nutrient Intake

It must be reiterated that current-day recall was not designed to determine the exact quantity of specific nutrients consumed. Good agreement between e-12HR and the reference methods (FFQ and DRs), with regard to a group of specific foods, does not imply good agreement between the nutrients that the food group provides an individual. This is due to the fact that specific nutrients may come from different food groups. For example, of the food groups considered in this study, legumes, chicken/turkey, fish, and red meat are all rich in proteins. Although, in general, we have observed good agreement between e-12HR and the reference methods considered by these food groups, this does not imply that e-12HR has the ability to determine the exact quantity of proteins consumed by an individual. This is because of other food groups also being rich in this nutrient (such as nuts, dairy products, or pasta), which were not considered in this study.

#### Format Used in Questionnaire

Full details on the format used in the questionnaires are available elsewhere [32,44,45]. In short, the e-12HR app is digital, and the FFQ and DRs are completed on paper. Paper formats are typically associated with errors such as unanswered questions, questions with multiple responses [7] (FFQ), and not registering the quantity consumed for some of the different food groups selected (DR) [55]. Despite the potential advantages of utilizing FFQs and DRs in digital format, in the end, it was decided to use paper formats in this study. The research team took into account that, on the one hand, evidence shows that data collected from smartphone apps and Web-based FFQs and DRs are comparable with data from paper formats [12-14,16,22,29,30,35,36,38,40,56], whereas on the other hand, due to the characteristics of this study, the potential disadvantages of developing FFQs and DRs in digital format could surpass the possible benefits. In fact, in this study, the paper-based FFQ and the DRs are very short and simple (they only contain 10 food groups), and the sample population is made up of students and employees at the Schools of Medicine and Pharmacy at the University of Seville, which is easily accessible for the research team. The simplicity of the paper-based FFQ and DRs minimized possible errors, the amount of paper used, problems with storage space, and costs associated with data conversion. These costs were minimal when compared with the potential costs of developing a Web-based or smartphone-based FFQ and DRs. Easy access to the sample made it possible to complete the paper FFQ in person, without the need for researchers or participants to travel or pay mailing costs [32,44,45].

#### Usability Rating Questionnaire for the Electronic 12-Hour Dietary Recall App

The majority of participants in this study reported that the e-12HR app was easy, brief, and interesting to complete; that they would be willing to complete the e-12HR app again; and that the task took 2 min or less per day to complete (see Tables 14 and 15). According to this latest piece of information from the study participants, the research team considered that the time necessary to complete the app is, normally, 2 min per day or less.

#### Sample Size

The sample size was established with the sample size software nQuery Advisor Version 7.0 (Statistical Solutions Ltd., One International Place, 100 Oliver Street, Boston MA, USA) [57]. For the SCC, alpha=.05, a value for the null hypothesis (lack of relation)=0.0, a value for the alternative hypothesis=0.5, and power of 90%. The sample size obtained was n=40.

The sample size reached (and amply surpassed) what was indicated in all of the strata except for one: *yes smoking* status with n=33 (see Table 1).

### Limitations

Limitations of this study included the fact that the sample used was extremely educated, which is a convenient sample (there is no random selection) and not representative of the population on the national level. In addition, as this is a convenience sample, made up of colleagues, students, and employees, the participants might have responded more favorably to the questions posed by the usability rating questionnaire for e-12HR. The small number of individuals in some of the subgroups is another limitation of the study, for example, smokers (n=33). Another limitation derives from the need to have a smartphone with an Android operating system. Access to these technologies is not universal and could exclude those students or employees with less purchasing power [44].

This method, as it was not designed to collect data on the exact quantity of specific nutrients consumed, does not allow for an analysis of the possible association between nutrients and chronic illnesses, rather only between categories of habitual consumption for food groups and risk of chronic illnesses.

Another limitation is that the soft drinks category does not differentiate between sugary drinks and artificially sugary drinks.

Ideally, validation studies should include the use of nutritional biomarkers, but currently, there are few biomarkers for specific foods [10,52,58,59] and they cannot measure habitual intake [52].

### Conclusions

For the whole sample of adults and for all strata thereof, the high correlation and good agreement between the e-12HR app and both reference methods (the FFQ and the 4 DRs), utilizing various procedures of statistical analysis, indicate the *relative validity* of the current-day recall for ranking the habitual intake of selected food groups.

For e-12HR versus FFQ, the inclusion of photographs to facilitate estimation of the servings consumed has not provided better correlation or agreement data between the methods, as the data obtained were similar to that of a previous study using an older version of the app without photographs.

The *relative validity* of current-day recall and the interesting features of e-12HR for users (the app is easy, brief, and interesting to complete [according to the usability rating questionnaire], and has photographs to assist with estimating servings consumed) as well as investigators (data collection is performed digitally, eliminating the need for investigators to later introduce the data manually; it is a self-reporting tool, not requiring interviewers; and overall research costs are greatly reduced), indicate that this method could be considered as a useful alternative to FFQs. This method (FFQ) is the most commonly implemented instrument in large-scale epidemiological and intervention studies, which do not require determining the complete diet nor the exact quantity consumed of a specific food group to analyze possible associations with risks for chronic diseases and for evaluating the effects of interventions.

## Appendix

Multimedia Appendix 1 Usability rating questionnaire for e-12HR.

Multimedia Appendix 2 Questionnaire used in the e-12HR app.

Multimedia Appendix 3 Screen capture of e-12HR.

Multimedia Appendix 4 Precoded dietary record.

Multimedia Appendix 5 Cross-classification analysis derived from the e-12HR app versus the food frequency questionnaire, and the e-12HR app versus the 4 dietary records.

Multimedia Appendix 6 Cases that did not fulfill the comparison criteria for categories of food group consumption derived from the e-12HR app versus the food frequency questionnaire, and from the e-12HR app versus the 4 dietary records.

## Abbreviations

BMI: body mass index
DR: dietary record
e-12HR: electronic 12-hour dietary recall
FFQ: food frequency questionnaire
SCC: Spearman correlation coefficient
