# Supplementary material for: Relative Validity of a Method Based on a Smartphone App (Electronic 12-Hour Dietary Recall) to Estimate Habitual Dietary Intake in Adults
Source: JMIR Mhealth Uhealth. 2019 Apr 11;7(4):e11531. doi: 10.2196/11531 (PMC6489347; doi:10.2196/11531)
Supplement: Multimedia Appendix 1 [file mhealth_v7i4e11531_app1.pdf]

**Multimedia Appendix 1.** Usability rating questionnaire for electronic 12-hour dietary recall app.

1. I found the e-12HR easy to complete:

Strongly agree.
Agree.
Neither agree nor disagree.
Disagree.
Strongly disagree.

2. I found the e-12HR too time consuming:

Strongly agree.
Agree.
Neither agree nor disagree.
Disagree.
Strongly disagree.

3. I found the e-12HR interesting to complete:

Strongly agree.
Agree.
Neither agree nor disagree.
Disagree.
Strongly disagree.

4. In the future, I would be willing to complete e-12HR again:

Strongly agree.
Agree.
Neither agree nor disagree.
Disagree.
Strongly disagree.

5. How much time was needed to complete the daily questionnaire on the app:

Less than 1 minute per day.
Approximately 1 minute per day.
Approximately 2 minutes per day.
Approximately 3 minutes per day.
Approximately 4 minutes per day.
5 minutes per day or more.
