# Supplementary material for: Relative Validity of a Method Based on a Smartphone App (Electronic 12-Hour Dietary Recall) to Estimate Habitual Dietary Intake in Adults
Source: JMIR Mhealth Uhealth. 2019 Apr 11;7(4):e11531. doi: 10.2196/11531 (PMC6489347; doi:10.2196/11531)
Supplement: Multimedia Appendix 2 [file mhealth_v7i4e11531_app2.pdf]

Multimedia Appendix 2. Questionnaire used in electronic 12-hour dietary recall app.

1. How many pieces of fruit have you eaten today?
2. How many portions of vegetables have you eaten today?
3. How many portions of legumes (lentils, garbanzos, beans, etc.) have you eaten today?
4. How many portions of chicken/turkey have you eaten today?
5. How many portions of fish have you eaten today?
6. How many portions of red meat (beef, pork, lamb) have you eaten today?
7. How many servings of soft drinks have you had today?
8. How many portions of commercially produced sweets (not home-made) (cookies/pastries) have you eaten today?
9. How many portions of prepared/frozen foods have you eaten today (croquettes, pizza, etc.)?
10. How many servings of beer have you consumed today?
