# Supplementary material for: Relative Validity of a Method Based on a Smartphone App (Electronic 12-Hour Dietary Recall) to Estimate Habitual Dietary Intake in Adults
Source: JMIR Mhealth Uhealth. 2019 Apr 11;7(4):e11531. doi: 10.2196/11531 (PMC6489347; doi:10.2196/11531)

Multimedia Appendix 3. Screen capture of the 12-hour dietary recall.

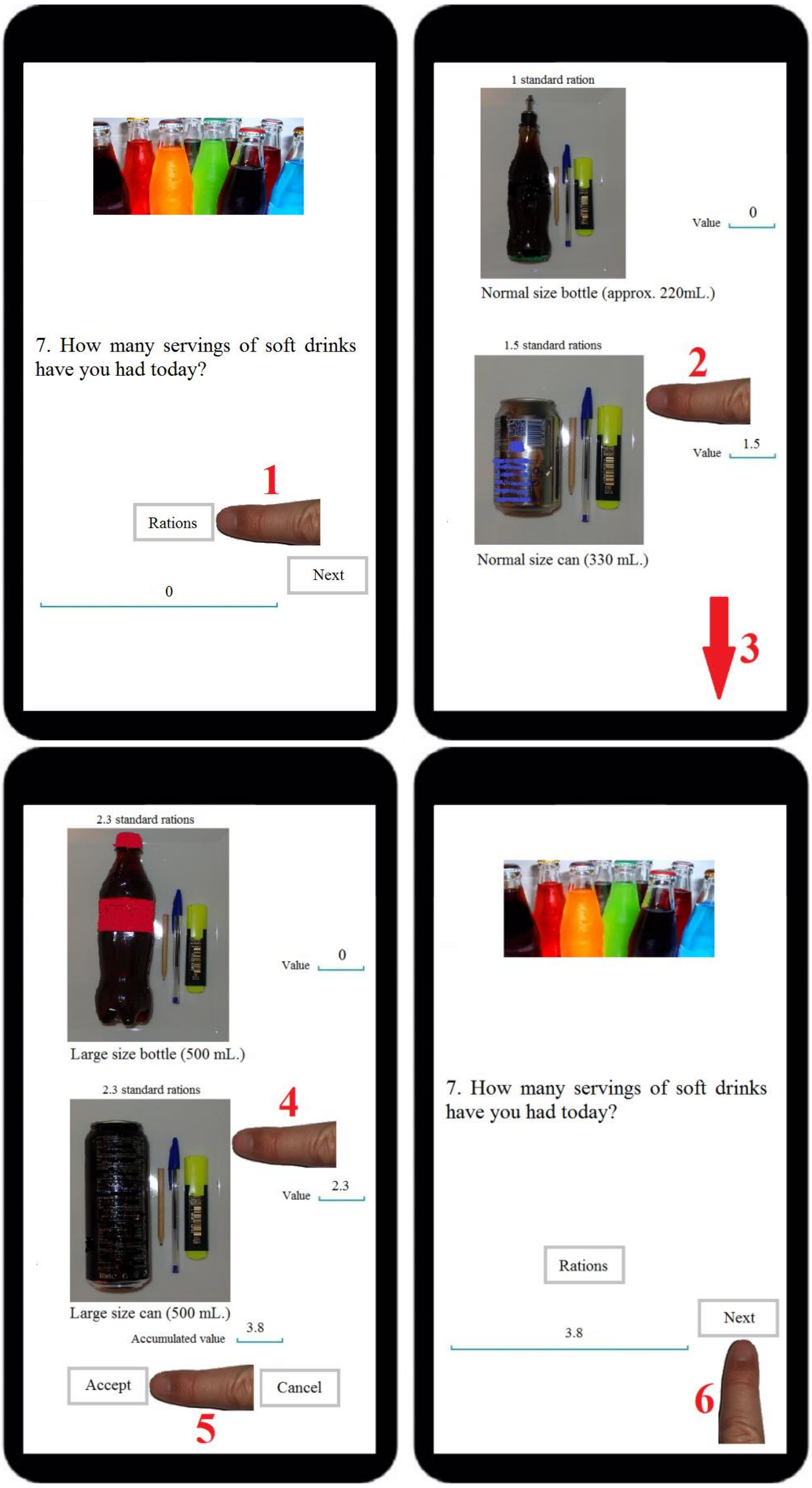

Supplement: Multimedia Appendix 3 [file mhealth_v7i4e11531_app3.pdf]
