# Supplementary material for: Relative Validity of a Method Based on a Smartphone App (Electronic 12-Hour Dietary Recall) to Estimate Habitual Dietary Intake in Adults
Source: JMIR Mhealth Uhealth. 2019 Apr 11;7(4):e11531. doi: 10.2196/11531 (PMC6489347; doi:10.2196/11531)
Supplement: Multimedia Appendix 4 [file mhealth_v7i4e11531_app4.pdf]

Multimedia Appendix 4. Pre-coded dietary record.

Personal alphanumeric code: __ __ __ __ __ __ __ __ __ __ __

Number of dietary record: 1 / 2 / 3 / 4

Day name: ____________________

Date (Day/Month/Year): __ __ / __ __ /__ __ __ __

	Morning (Before 12am)	Afternoon (12am-6pm)	Evening and night (6pm-12pm)
Fruits **(g.)**<br>Fruit juice **(mL.)**			
Vegetables **(g.)**			
Legumes **(g.)**			
Chicken/turkey **(g.)**			
Fish **(g.)**			
Red meat **(g.)**			
Soft drinks **(mL.)**			
Sweets **(g.)**			
Prepared foods **(g.)**			
Beer **(mL.)**			

Record the consumption of each of the foods in grams (g.) and drinks in millilitres (mL.) in the time in which they were consumed.
