# Supplementary material for: Relative Validity of a Method Based on a Smartphone App (Electronic 12-Hour Dietary Recall) to Estimate Habitual Dietary Intake in Adults
Source: JMIR Mhealth Uhealth. 2019 Apr 11;7(4):e11531. doi: 10.2196/11531 (PMC6489347; doi:10.2196/11531)
Supplement: Multimedia Appendix 5 [file mhealth_v7i4e11531_app5.pdf]

Multimedia Appendix 5. Cross-classification analysis derived from electronic 12-hour dietary recall (e-12HR) app versus the food frequency questionnaire and from e-12HR app versus the 4 dietary records.

	Comparison e-12HR vs FFQ						
	All	Age (years)		Gender		Occupation	
		< 25	≥ 25	Females	Males	Students	Employees
	Fruit						
Total	203 (100)	82 (100)	121 (100)	115 (100)	88 (100)	87 (100)	116 (100)
Exact agreement[a] (%)	112 (55.2)	43 (52.4)	69 (57.0)	67 (58.3)	45 (51.1)	47 (54.0)	65 (56.0)
Exact agreement + adjacent[b] (%)	182 (89.7)	71 (86.6)	111 (91.7)	101 (87.8)	81 (92.0)	76 (87.4)	106 (91.4)
Extreme disagreement[c] (%)	0 (0.0)	0 (0.0)	0 (0.0)	0 (0.0)	0 (0.0)	0 (0.0)	0 (0.0)
	Vegetables						
Exact agreement	99 (48.8)	41 (50.0)	58 (47.9)	64 (55.7)	35 (39.8)	43 (49.4)	56 (48.3)
Exact agreement + adjacent	178 (87.7)	74 (90.2)	104 (86.0)	103 (89.6)	75 (85.2)	80 (92.0)	98 (84.5)
Extreme disagreement	0 (0.0)	0 (0.0)	0 (0.0)	0 (0.0)	0 (0.0)	0 (0.0)	0 (0.0)
	Legumes						
Exact agreement	119 (58.6)	46 (56.1)	73 (60.3)	71 (61.7)	48 (54.5)	49 (56.3)	70 (60.3)
Exact agreement + adjacent	196 (96.6)	78 (95.1)	119 (98.3)	110 (95.7)	87 (98.9)	84 (96.6)	113 (97.4)
Extreme disagreement	0 (0.0)	0 (0.0)	0 (0.0)	0 (0.0)	0 (0.0)	0 (0.0)	0 (0.0)
	Chicken/turkey						
Exact agreement	84 (41.4)	30 (36.6)	54 (44.6)	43 (37.4)	41 (46.6)	33 (37.9)	51 (44.0)
Exact agreement + adjacent	180 (88.7)	70 (85.4)	110 (90.9)	99 (86.1)	81 (92.0)	75 (86.2)	105 (90.5)
Extreme disagreement	0 (0.0)	0 (0.0)	0 (0.0)	0 (0.0)	0 (0.0)	0 (0.0)	0 (0.0)
	Fish						
Exact agreement	85 (41.9)	38 (46.3)	47 (38.8)	45 (39.1)	40 (45.5)	42 (48.3)	43 (37.1)
Exact agreement + adjacent	175 (86.2)	72 (87.8)	103 (85.1)	101 (87.8)	74 (84.1)	79 (90.8)	96 (82.8)
Extreme disagreement	0 (0.0)	0 (0.0)	0 (0.0)	0 (0.0)	0 (0.0)	0 (0.0)	0 (0.0)
	Red meat						
Exact agreement	108 (53.2)	49 (59.8)	59 (48.8)	66 (57.4)	42 (47.7)	51 (58.6)	57 (49.1)
Exact agreement + adjacent	192 (94.6)	78 (95.1)	114 (94.2)	111 (96.5)	81 (92.0)	83 (95.4)	109 (94.0)
Extreme disagreement	0 (0.0)	0 (0.0)	0 (0.0)	0 (0.0)	0 (0.0)	0 (0.0)	0 (0.0)
	Soft drinks						
Exact agreement	108 (53.2)	42 (51.2)	66 (54.5)	67 (58.3)	41 (46.6)	46 (52.9)	62 (53.4)
Exact agreement + adjacent	180 (88.7)	76 (92.7)	104 (86.0)	104 (90.4)	76 (86.4)	83 (95.4)	97 (83.6)
Extreme disagreement	0 (0.0)	0 (0.0)	0 (0.0)	0 (0.0)	0 (0.0)	0 (0.0)	0 (0.0)
	Sweets						
Exact agreement	85 (41.9)	33 (40.2)	52 (43.0)	50 (45.3)	35 (39.8)	35 (40.2)	50 (43.1)
Exact agreement + adjacent	174 (85.7)	70 (85.4)	104 (86.0)	101 (87.8)	73 (83.0)	74 (85.1)	100 (86.2)
Extreme disagreement	0 (0.0)	0 (0.0)	0 (0.0)	0 (0.0)	0 (0.0)	0 (0.0)	0 (0.0)
	Prepared foods						
Exact agreement	104 (51.2)	37 (45.1)	67 (55.4)	65 (56.5)	39 (44.3)	41 (47.1)	63 (54.3)
Exact agreement + adjacent	194 (95.6)	79 (96.3)	115 (95.0)	111 (96.5)	83 (94.3)	84 (96.6)	110 (94.8)
Extreme disagreement	0 (0.0)	0 (0.0)	0 (0.0)	0 (0.0)	0 (0.0)	0 (0.0)	0 (0.0)
	Beer						
Exact agreement	126 (62.1)	58 (70.7)	68 (56.2)	78 (67.8)	48 (54.5)	61 (70.1)	65 (56.0)
Exact agreement + adjacent	181 (89.2)	76 (92.7)	105 (86.8)	104 (90.4)	77 (87.5)	80 (92.0)	101 (87.1)
Extreme disagreement	0 (0.0)	0 (0.0)	0 (0.0)	0 (0.0)	0 (0.0)	0 (0.0)	0 (0.0)
	Average						
Exact agreement	(50.7)	(50.9)	(50.7)	(53.6)	(47.0)	(51.5)	(50.2)
Exact agreement + adjacent	(90.2)	(90.7)	(90.0)	(90.9)	(89.5)	(91.8)	(89.2)
Extreme disagreement	(0.0)	(0.0)	(0.0)	(0.0)	(0.0)	(0.0)	(0.0)
		Smoking		Physical activity (minutes/week)		BMI (kg/m^2)	
		No	Yes	≥ 150	< 150	< 25	≥ 25
	Fruit						
Total		170 (100)	33 (100)	135 (100)	68 (100)	134 (100)	69 (100)
Exact agreement		94 (55.3)	18 (54.5)	74 (54.8)	38 (55.9)	74 (55.2)	38 (55.1)

Exact agreement + adjacent		152 (89.4)	30 (90.9)	120 (88.9)	62 (91.2)	118 (88.1)	64 (92.8)
Extreme disagreement		0 (0.0)	0 (0.0)	0 (0.0)	0 (0.0)	0 (0.0)	0 (0.0)
	Vegetables						
Exact agreement		84 (49.4)	15 (45.5)	68 (50.4)	31 (45.6)	64 (47.8)	35 (50.7)
Exact agreement + adjacent		150 (88.2)	28 (84.8)	119 (88.1)	59 (86.8)	118 (88.1)	60 (87.0)
Extreme disagreement		0 (0.0)	0 (0.0)	0 (0.0)	0 (0.0)	0 (0.0)	0 (0.0)
	Legumes						
Exact agreement		97 (57.1)	22 (66.7)	79 (58.5)	40 (58.8)	78 (58.2)	41 (59.4)
Exact agreement + adjacent		164 (96.5)	33 (100.0)	132 (97.8)	65 (95.6)	129 (96.3)	68 (98.6)
Extreme disagreement		0 (0.0)	0 (0.0)	0 (0.0)	0 (0.0)	0 (0.0)	0 (0.0)
	Chicken/turkey						
Exact agreement		75 (44.1)	9 (27.3)	53 (39.3)	31 (45.6)	61 (45.5)	23 (33.3)
Exact agreement + adjacent		153 (90.0)	27 (81.8)	117 (86.7)	63 (92.6)	120 (89.6)	60 (87.0)
Extreme disagreement		0 (0.0)	0 (0.0)	0 (0.0)	0 (0.0)	0 (0.0)	0 (0.0)
	Fish						
Exact agreement		70 (41.2)	15 (45.5)	57 (42.2)	28 (41.2)	56 (41.8)	29 (42.0)
Exact agreement + adjacent		145 (85.3)	30 (90.9)	115 (85.2)	60 (88.2)	119 (88.8)	56 (81.2)
Extreme disagreement		0 (0.0)	0 (0.0)	0 (0.0)	0 (0.0)	0 (0.0)	0 (0.0)
	Red meat						
Exact agreement		90 (52.9)	18 (54.5)	67 (49.6)	41 (60.3)	72 (53.7)	36 (52.2)
Exact agreement + adjacent		160 (94.1)	32 (97.0)	128 (94.8)	64 (94.1)	127 (94.8)	65 (94.2)
Extreme disagreement		0 (0.0)	0 (0.0)	0 (0.0)	0 (0.0)	0 (0.0)	0 (0.0)
	Soft drinks						
Exact agreement		90 (52.9)	18 (54.5)	74 (54.8)	34 (50.0)	78 (58.2)	32 (46.4)
Exact agreement + adjacent		152 (89.4)	28 (84.8)	120 (88.9)	60 (88.2)	126 (94.0)	56 (81.2)
Extreme disagreement		0 (0.0)	0 (0.0)	0 (0.0)	0 (0.0)	0 (0.0)	0 (0.0)
	Sweets						
Exact agreement		68 (40.0)	17 (51.5)	61 (45.2)	24 (35.3)	55 (41.0)	30 (43.5)
Exact agreement + adjacent		146 (85.9)	29 (87.9)	119 (88.1)	55 (80.9)	116 (86.6)	58 (84.1)
Extreme disagreement		0 (0.0)	0 (0.0)	0 (0.0)	0 (0.0)	0 (0.0)	0 (0.0)
	Prepared foods						
Exact agreement		90 (52.9)	14 (42.4)	73 (54.1)	31 (45.6)	65 (48.5)	39 (56.5)
Exact agreement + adjacent		163 (95.9)	31 (93.9)	130 (96.3)	64 (94.1)	129 (96.3)	65 (94.2)
Extreme disagreement		0 (0.0)	0 (0.0)	0 (0.0)	0 (0.0)	0 (0.0)	0 (0.0)
	Beer						
Exact agreement		108 (63.5)	18 (54.5)	88 (65.2)	38 (55.9)	87 (64.9)	39 (56.5)
Exact agreement + adjacent		152 (89.4)	29 (87.9)	121 (89.6)	60 (88.2)	122 (91.0)	59 (85.5)
Extreme disagreement		0 (0.0)	0 (0.0)	0 (0.0)	0 (0.0)	0 (0.0)	0 (0.0)
	Average						
Exact agreement		(50.9)	(49.7)	(51.4)	(49.4)	(51.5)	(49.6)
Exact agreement + adjacent		(90.4)	(90.0)	(90.4)	(90.0)	(91.3)	(88.6)
Extreme disagreement		(0.0)	(0.0)	(0.0)	(0.0)	(0.0)	(0.0)
	Comparisone-12HR vs DRs						
	All	Age (years)		Gender		Occupation	
		< 25	≥ 25	Females	Males	Students	Employees
	Fruit						
Total	203 (100)	82 (100)	121 (100)	115 (100)	88 (100)	87 (100)	116 (100)
Exact agreement	130 (64.0)	55 (67.1)	75 (62.0)	76 (66.1)	54 (61.4)	57 (65.5)	73 (62.9)
Exact agreement + adjacent	184 (90.6)	76 (92.7)	108 (89.3)	105 (91.3)	79 (89.8)	81 (93.1)	103 (88.8)
Extreme disagreement	0 (0.0)	0 (0.0)	0 (0.0)	0 (0.0)	0 (0.0)	0 (0.0)	0 (0.0)
	Vegetables						
Exact agreement	94 (46.3)	34 (41.5)	60 (49.6)	55 (47.8)	39 (44.3)	34 (39.1)	60 (51.7)
Exact agreement + adjacent	186 (91.6)	76 (92.7)	110 (90.9)	107 (93.0)	79 (89.8)	79 (90.8)	107 (92.2)
Extreme disagreement	0 (0.0)	0 (0.0)	0 (0.0)	0 (0.0)	0 (0.0)	0 (0.0)	0 (0.0)
	Legumes						
Exact agreement	104 (51.2)	43 (52.4)	61 (50.4)	56 (48.7)	48 (54.5)	48 (55.2)	56 (48.3)
Exact agreement + adjacent	191 (94.1)	77 (93.9)	114 (94.2)	107 (93.0)	84 (95.5)	83 (95.4)	108 (93.1)
Extreme disagreement	0 (0.0)	0 (0.0)	0 (0.0)	0 (0.0)	0 (0.0)	0 (0.0)	0 (0.0)

	Chicken/turkey						
Exact agreement	79 (38.9)	26 (31.7)	53 (43.8)	44 (38.3)	35 (39.8)	27 (31.0)	52 (44.8)
Exact agreement + adjacent	171 (84.2)	72 (87.8)	99 (81.8)	102 (88.7)	69 (78.4)	76 (87.4)	95 (81.9)
Extreme disagreement	0 (0.0)	0 (0.0)	0 (0.0)	0 (0.0)	0 (0.0)	0 (0.0)	0 (0.0)
	Fish						
Exact agreement	70 (34.5)	28 (34.1)	42 (34.7)	41 (35.6)	29 (33.0)	28 (32.2)	42 (36.2)
Exact agreement + adjacent	170 (83.7)	72 (87.8)	98 (81.0)	98 (85.2)	72 (81.8)	77 (88.5)	93 (80.2)
Extreme disagreement	0 (0.0)	0 (0.0)	0 (0.0)	0 (0.0)	0 (0.0)	0 (0.0)	0 (0.0)
	Red meat						
Exact agreement	82 (40.4)	32 (39.0)	50 (41.3)	52 (45.2)	30 (34.1)	34 (39.1)	48 (41.4)
Exact agreement + adjacent	175 (86.2)	72 (87.8)	103 (85.1)	104 (90.4)	71 (80.7)	77 (88.5)	98 (84.5)
Extreme disagreement	0 (0.0)	0 (0.0)	0 (0.0)	0 (0.0)	0 (0.0)	0 (0.0)	0 (0.0)
	Soft drinks						
Exact agreement	115 (56.7)	48 (58.5)	67 (55.4)	71 (61.7)	44 (50.0)	50 (57.5)	65 (56.0)
Exact agreement + adjacent	176 (86.7)	67 (81.7)	109 (90.1)	101 (87.8)	75 (85.2)	73 (83.9)	103 (88.8)
Extreme disagreement	0 (0.0)	0 (0.0)	0 (0.0)	0 (0.0)	0 (0.0)	0 (0.0)	0 (0.0)
	Sweets						
Exact agreement	98 (48.3)	34 (41.5)	64 (52.9)	57 (49.6)	41 (46.6)	35 (40.2)	63 (54.3)
Exact agreement + adjacent	179 (88.2)	76 (92.7)	103 (85.1)	104 (90.4)	75 (85.2)	78 (89.6)	101 (87.1)
Extreme disagreement	0 (0.0)	0 (0.0)	0 (0.0)	0 (0.0)	0 (0.0)	0 (0.0)	0 (0.0)
	Prepared foods						
Exact agreement	104 (51.2)	35 (42.7)	69 (57.0)	62 (53.9)	42 (47.7)	39 (44.8)	65 (56.0)
Exact agreement + adjacent	182 (89.7)	72 (87.8)	110 (90.9)	99 (86.1)	83 (94.3)	77 (88.5)	105 (90.5)
Extreme disagreement	0 (0.0)	0 (0.0)	0 (0.0)	0 (0.0)	0 (0.0)	0 (0.0)	0 (0.0)
	Beer						
Exact agreement	139 (68.5)	56 (68.3)	83 (68.6)	87 (75.7)	52 (59.1)	58 (66.7)	81 (69.8)
Exact agreement + adjacent	177 (87.2)	72 (87.8)	105 (86.8)	106 (92.2)	71 (80.7)	75 (86.2)	102 (87.9)
Extreme disagreement	0 (0.0)	0 (0.0)	0 (0.0)	0 (0.0)	0 (0.0)	0 (0.0)	0 (0.0)
	Average						
Exact agreement	(50.0)	(47.7)	(51.6)	(52.3)	(47.0)	(47.1)	(52.2)
Exact agreement + adjacent	(88.2)	(89.3)	(87.5)	(89.8)	(86.1)	(89.2)	(87.5)
Extreme disagreement	(0.0)	(0.0)	(0.0)	(0.0)	(0.0)	(0.0)	(0.0)
		Smoking		Physical activity (minutes/week)		BMI (kg/m^2)	
		No	Yes	≥ 150	< 150	< 25	≥ 25
	Fruit						
Total		170 (100)	33 (100)	135 (100)	68 (100)	134 (100)	69 (100)
Exact agreement		113 (66.5)	17 (51.5)	83 (61.5)	47 (69.1)	87 (64.9)	43 (62.3)
Exact agreement + adjacent		153 (90.0)	31 (93.9)	120 (88.9)	64 (94.1)	121 (90.3)	63 (91.3)
Extreme disagreement		0 (0.0)	0 (0.0)	0 (0.0)	0 (0.0)	0 (0.0)	0 (0.0)
	Vegetables						
Exact agreement		80 (47.1)	14 (42.4)	67 (49.6)	27 (39.7)	57 (42.5)	37 (53.6)
Exact agreement + adjacent		158 (92.9)	28 (84.8)	126 (93.3)	60 (88.2)	125 (93.3)	61 (88.4)
Extreme disagreement		0 (0.0)	0 (0.0)	0 (0.0)	0 (0.0)	0 (0.0)	0 (0.0)
	Legumes						
Exact agreement		88 (51.8)	16 (48.5)	69 (51.1)	35 (51.5)	69 (51.5)	35 (50.7)
Exact agreement + adjacent		159 (93.5)	32 (97.0)	127 (94.1)	64 (94.1)	124 (92.5)	67 (97.1)
Extreme disagreement		0 (0.0)	0 (0.0)	0 (0.0)	0 (0.0)	0 (0.0)	0 (0.0)
	Chicken/turkey						
Exact agreement		65 (38.2)	14 (42.4)	55 (40.7)	24 (35.3)	55 (41.0)	24 (34.8)
Exact agreement + adjacent		143 (84.1)	28 (84.8)	112 (83.0)	59 (86.8)	115 (85.8)	56 (81.2)
Extreme disagreement		0 (0.0)	0 (0.0)	0 (0.0)	0 (0.0)	0 (0.0)	0 (0.0)
	Fish						
Exact agreement		58 (34.1)	12 (36.4)	45 (33.3)	25 (36.8)	53 (39.6)	17 (24.6)
Exact agreement + adjacent		139 (81.8)	31 (93.9)	113 (83.7)	57 (83.8)	118 (88.1)	52 (75.4)
Extreme disagreement		0 (0.0)	0 (0.0)	0 (0.0)	0 (0.0)	0 (0.0)	0 (0.0)
	Red meat						
Exact agreement		69 (40.6)	13 (39.4)	59 (43.7)	23 (33.8)	57 (42.5)	25 (36.2)

Exact agreement + adjacent		147 (86.5)	28 (84.8)	118 (87.4)	57 (83.8)	115 (85.8)	60 (87.0)
Extreme disagreement		0 (0.0)	0 (0.0)	0 (0.0)	0 (0.0)	0 (0.0)	0 (0.0)
	Soft drinks						
Exact agreement		96 (56.5)	19 (57.6)	75 (55.6)	40 (58.8)	84 (62.7)	31 (44.9)
Exact agreement + adjacent		145 (85.3)	31 (93.9)	116 (85.9)	60 (88.2)	117 (87.3)	59 (85.5)
Extreme disagreement		0 (0.0)	0 (0.0)	0 (0.0)	0 (0.0)	0 (0.0)	0 (0.0)
	Sweets						
Exact agreement		81 (47.6)	17 (51.5)	66 (48.9)	32 (47.1)	66 (49.3)	32 (46.4)
Exact agreement + adjacent		150 (88.2)	29 (87.9)	120 (88.9)	59 (86.8)	120 (89.6)	59 (85.5)
Extreme disagreement		0 (0.0)	0 (0.0)	0 (0.0)	0 (0.0)	0 (0.0)	0 (0.0)
	Prepared foods						
Exact agreement		88 (51.8)	16 (48.5)	75 (55.6)	29 (42.6)	64 (47.8)	40 (58.0)
Exact agreement + adjacent		153 (90.0)	29 (87.9)	121 (89.6)	61 (89.7)	119 (88.8)	63 (91.3)
Extreme disagreement		0 (0.0)	0 (0.0)	0 (0.0)	0 (0.0)	0 (0.0)	0 (0.0)
	Beer						
Exact agreement		121 (70.8)	18 (54.5)	94 (69.6)	45 (66.2)	92 (68.7)	47 (68.1)
Exact agreement + adjacent		150 (88.2)	27 (81.8)	117 (86.7)	60 (88.2)	118 (88.1)	59 (85.5)
Extreme disagreement		0 (0.0)	0 (0.0)	0 (0.0)	0 (0.0)	0 (0.0)	0 (0.0)
	Average						
Exact agreement		(50.5)	(47.3)	(51.0)	(48.1)	(51.0)	(48.0)
Exact agreement + adjacent		(88.1)	(89.1)	(88.1)	(88.4)	(89.0)	(86.8)
Extreme disagreement		(0.0)	(0.0)	(0.0)	(0.0)	(0.0)	(0.0)

[a]Exact agreement: cases cross-classified into the same category.
[b]Exact agreement + adjacent: cases cross-classified into the same or adjacent category.
[c]Extreme disagreement: cases cross-classified into extreme categories.
