# Supplementary material for: Relative Validity of a Method Based on a Smartphone App (Electronic 12-Hour Dietary Recall) to Estimate Habitual Dietary Intake in Adults
Source: JMIR Mhealth Uhealth. 2019 Apr 11;7(4):e11531. doi: 10.2196/11531 (PMC6489347; doi:10.2196/11531)
Supplement: Multimedia Appendix 6 [file mhealth_v7i4e11531_app6.pdf]

Multimedia Appendix 6. Cases that did not fulfill the comparison criteria for categories of food group consumption derived from electronic 12-hour dietary recall (e-12HR) app versus the food frequency questionnaire, and from e-12HR app versus the 4 dietary records.

Comparison of electronic 12-hour dietary recall app versus food frequency questionnaire					
Legumes					
Spearman correlation coefficients		Cross-classification (exact agreement)		Weighted kappa	
Stratus	Value	Stratus	n/N (%)	Stratus	Value
All	0.48	-[a]	-	All	0.37
<25 years	0.47	-	-	<25 years	0.35
Males	0.46	-	-	≥25 years	0.38
Employees	0.49	-	-	Females	0.38
No smoking	0.49	-	-	Males	0.34
Yes smoking	0.41	-	-	Students	0.38
≥150 min/week	0.46	-	-	Employees	0.37
<25 kg/m^2	0.47	-	-	No smoking	0.37
-	-	-	-	Yes smoking	0.33
-	-	-	-	≥150 min/week	0.37
-	-	-	-	<150 min/week	0.35
-	-	-	-	<25 kg/m^2	0.35
-	-	-	-	≥25 kg/m^2	0.40
Chicken and turkey					
Spearman correlation coefficients		Cross-classification (exact agreement)		Weighted kappa	
Stratus	Value	Stratus	n/N (%)	Stratus	Value
<25 years	0.49	Yes smoking	9/33 (27)	All	0.40
Yes smoking	0.38	-	-	<25 years	0.32
-	-	-	-	Females	0.34
-	-	-	-	Students	0.34
-	-	-	-	Yes smoking	0.26
-	-	-	-	≥150 min/week	0.39
-	-	-	-	≥25 kg/m^2	0.31
Fish					
Spearman correlation coefficients		Cross-classification (exact agreement)		Weighted kappa	
Stratus	Value	Stratus	n/N (%)	Stratus	Value
≥25 years	0.48	-	-	All	0.30

Employees	0.44	-	-	<25 years	0.37
≥25 kg/m^2	0.42	-	-	≥25 years	0.26
-		-	-	Females	0.29
-	-	-	-	Males	0.32
-	-	-	-	Employees	0.22
-	-	-	-	No smoking	0.30
-	-	-	-	Yes smoking	0.34
-	-	-	-	≥150 min/week	0.30
-	-	-	-	<150 min/week	0.30
-	-	-	-	<25 kg/m^2	0.34
-	-	-	-	≥25 kg/m^2	0.23
Red meat					
Spearman correlation coefficients		Cross-classification (exact agreement)		Weighted kappa	
Stratus	Value	Stratus	n/N (%)	Stratus	Value
-	-	-	-	≥25 years	0.38
-	-	-	-	Males	0.37
-	-	-	-	Employees	0.39
Prepared foods					
Spearman correlation coefficients		Cross-classification (exact agreement)		Weighted kappa	
Stratus	Value	Stratus	n/N (%)	Stratus	Value
Yes smoking	0.44	-	-	All	0.40
-	-	-	-	<25 years	0.38
-	-	-	-	≥25years	0.40
-	-	-	-	Females	0.40
-	-	-	-	Males	0.40
-	-	-	-	Employees	0.36
-	-	-	-	Yes smoking	0.22
-	-	-	-	<150 min/week	0.37
-	-		-	<25 kg/m^2	0.39
Comparison of electronic 12-hour dietary recall app versus dietary records					
Legumes					
Spearman correlation coefficients		Cross-classification (exact agreement)		Weighted kappa	
Stratus	Value	Stratus	n/N (%)	Stratus	Value
All	0.40	-	-	All	0.29
<25 years	0.40	-	-	<25 years	0.28
≥25 years	0.43	-	-	≥25 years	0.30

Females	0.34	-	-	Females	0.21
Males	0.47	-	-	Males	0.38
Students	0.46	-	-	Students	0.34
Employees	0.39	-	-	Employees	0.26
No smoking	0.42	-	-	No smoking	0.31
Yes smoking	0.29[b]	-	-	Yes smoking	0.18[b]
≥150 min/week	0.40	-	-	≥150 min/week	0.32
<150 min/week	0.39	-	-	<150 min/week	0.22
<25 kg/m^2	0.38	-	-	<25 kg/m^2	0.26
≥25 kg/m^2	0.43	-	-	≥25 kg/m^2	0.34
Chicken and turkey					
Spearman correlation coefficients		Cross-classification (exact agreement)		Weighted kappa	
Stratus	Value	Stratus	n/N (%)	Stratus	Value
-	-	<25 years	26/82 (32)	<25 years	0.36
-	-	Students	27/87 (31)	Students	0.36
-	-	≥25 kg/m^2	24/69 (35)	≥25 kg/m^2	0.39
Fish					
Spearman correlation coefficients		Cross-classification (exact agreement)		Weighted kappa	
Stratus	Value	Stratus	n/N (%)	Stratus	Value
≥25 years	0.49	All	70/203 (34.5)	All	0.31
<150 min/week	0.46	<25 years	28/82 (34)	<25 years	0.29
≥25 kg/m^2	0.33	≥25 years	42/121 (34.7)	≥25 years	0.29
-	-	Males	29/88 (33)	Females	0.29
-	-	Students	28/87 (32)	Males	0.33
-	-	No smoking	58/170 (34.1)	Students	0.26
-	-	≥150 min/week	45/135 (33.3)	Employees	0.31
-	-	≥25 kg/m^2	17/69 (25)	No smoking	0.30
-	-	-	-	Yes smoking	0.34
-	-	-	-	≥150 min/week	0.32
-	-	-	-	<150 min/week	0.25
-	-	-	-	<25 kg/m^2	0.39
-	-	-	-	≥25 kg/m^2	0.16
Red meat					
Spearman correlation coefficients		Cross-classification (exact agreement)		Weighted kappa	
Stratus	Value	Stratus	n/N (%)	Stratus	Value
Females	0.45	Males	30/88 (34)	All	0.34

Yes smoking	0.31[b]	<150 min/week	23/68 (34)	<25 years	0.34
<150 min/week	0.39	-	-	≥25 years	0.35
-		-	-	Females	0.31
-	-	-	-	Males	0.32
-	-	-	-	Students	0.34
-	-	-	-	Employees	0.34
-	-	-	-	No smoking	0.36
-	-	-	-	Yes smoking	0.19
-	-	-	-	≥150 min/week	0.39
-	-	-	-	<150 min/week	0.24
-	-	-	-	<25 kg/m^2	0.35
-	-	-	-	≥25 kg/m^2	0.33

In all cases, the criteria comparisons were fulfilled for *Exact agreement + adjacent* and *Extreme disagreement*.

[a]Not applicable.

[b]*P*<.05
